# Targeted metabolomics for bioprocessing

**DOI:** 10.1186/1753-6561-5-S8-P27

**Published:** 2011-11-22

**Authors:** Denise Sonntag, Francesca M Scandurra, Torben Friedrich, Michael Urban, Klaus M Weinberger

**Affiliations:** 1 BIOCRATES Life Sciences AG, Innsbruck, Austria

## Background

Bioprocesses like the cell-based production of biologicals, i.e. mainly recombinant proteins and monoclonal antibodies, require optimal culture conditions to obtain a high yield of quality products. The performance of a bioreactor highly depends on the cell characteristics as well as on the composition of the cell culture medium and the process conditions. As the metabolic activity of the cells is very high during fermentation, the external and internal metabolite compositions vary tremendously throughout the process. The quantification of a wide range of metabolic substrates and products is a prerequisite to understand and optimize the underlying cell-based activities. Furthermore, metabolite quantification reveals the composition of biologically derived cell culture supplements, thus serving as a tool to monitor supplement quality or providing the base for the formulation of a chemically defined medium supplement.

## Materials and methods

Targeted metabolomics was carried out using a mass spectrometry-based platform. Analyses were performed with commercially available KIT plates that allow the simultaneous quantification of 180 metabolites [1]. The fully automated assay is based on PITC (phenylisothiocyanate)-derivatization in the presence of internal standards followed by FIA-MS/MS (for acylcarnitines, lipids, hexoses) as well as LC-MS/MS (amino acids and biogenic amines) using a AB SCIEX 4000 QTrap mass spectrometer in the multiple reaction monitoring detection mode with electrospray ionization. On the same instrument, a validated HPLC-MS/MS quantification method is used for the analysis of energy metabolism intermediates. The concentrations of individual fatty acids are determined as their corresponding methyl ester derivatives (FAME’s) using GC-MS on an Agilent 7890 GC/5795 MSD instrument after derivatization. Vitamins were determined using LC-ESI-MS/MS technique after their pre-separation into two fractions (water- and fat-soluble vitamins).

## Results

**Figure 1:**
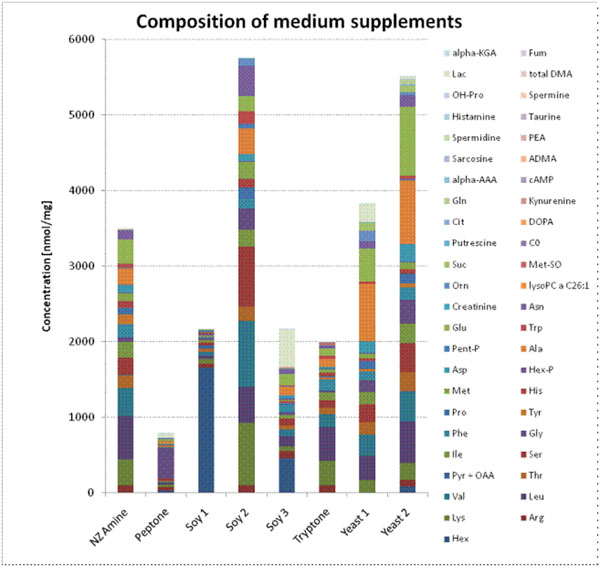
Comparison of the metabolite spectrum of commercially available cell culture media supplements derived from biological sources.

The metabolomics approach to bioprocess monitoring made it possible to determine the concentration of a multitude of analytes from several metabolite classes in a sample- and time-efficient way by using small sample volumes and a multi-assay strategy. The analyte portfolio went beyond routinely determined culture media components, as it covered proteinogenic and non-proteinogenic amino acids, e.g. ornithine and citrulline, and intermediates of the energy metabolism, e.g. lactate, hexoses, succinate, as well as acylcarnitines, biogenic amines, glycerophospholipids, sphingomyelins, fatty acids, and vitamins (Figure 1).

## Conclusions

Targeted metabolomics comprises a rapid and comprehensive method to determine the composition of cell culture supplements of biological origin.

The method is also well suited to rapidly characterize fermentational processes metabolically by monitoring changes in medium composition and cellular metabolite pools. The received quantitative data can be directly related to growth, vitality, and productivity of the cell.
